# Sociodemographic and behavioral characteristics of older people with HIV in Almaty City, Kazakhstan: A cross-sectional study

**DOI:** 10.1371/journal.pone.0328821

**Published:** 2025-08-05

**Authors:** Nursultan Nurzhigitov, Deborah Gustafson, Zhamilya Nugmanova, Alfiya Denebayeva, Aigerim Alimbekova, Gulnara Nugumanova, Gulmira Kalzhanbayeva, Ademi Sarsembiyeva, Jack DeHovitz

**Affiliations:** 1 HIV Infection and Infection Control Division, Department of Epidemiology, SPH, Asfendiyarov Kazakh National Medical University (KNMU), Almaty, Kazakhstan; 2 Department of Neurology, State University of New York Downstate Health Sciences University, Brooklyn, New York, United States of America; 3 Almaty AIDS Center, Almaty, Kazakhstan; 4 College of Medicine, State University of New York Downstate Health Sciences University, Brooklyn, New York, United States of America; University of Torino, ITALY

## Abstract

**Introduction:**

Globally, there are increasing numbers of people with HIV (PWH) age ≥ 40 years (y). Despite this, there are few studies of older PWH in low- and middle-income countries, and no studies in Kazakhstan. Aging with HIV influences HIV treatment response and is associated with the occurrence of non-communicable diseases.

**Methods and materials:**

A cross-sectional study of PWH ≥ 40y was conducted at the Almaty City AIDS Center in Kazakhstan. Biological, sociodemographic, health behavior, medical history, mental and cognitive health, quality of life, and HIV factors were measured and characterized.

**Results:**

The study enrolled and interviewed a clinical sample of 150 PWH ≥ 40y. Males (sex at birth) comprised 54.7% (N = 82); average age 51.6y, + /-10.4y; 54.7% aged 40-49y; maximum age 75y. Females (sex at birth) were younger (47.5y) than males (56.8y) (p = 0.020). Level of education differed by sex (p = 0.001). Females with HIV were more likely than males to be unemployed (33.8% versus 24.5%, respectively, p < 0.001). Among all PWH, 4% were disabled, 8.7% retired, 37.3% were in an official or civil marriage and 60.7% lived in their own home. More males consumed alcohol than females (32.9% versus 1.3%, respectively) (p = 0.001). Drug use was higher among males (25.6%) compared to females (8.8%) (p = 0.050); 91.4% of males versus 64.7% of females smoked ≥100 cigarettes in their lifetime, and 76.8% of males versus 48.5% of females smoked cigarettes currently (p = 0.001). HIV viral load among males was higher (p = 0.050) and CD4 + count, lower among males (p = 0.004) compared to females. Physical and physiological health, environmental and social relationships among males and females were similar.

**Conclusions:**

The majority of PWH were of working age, employed, and reported good quality of life, however, sex differences were observed. Future prospective studies are recommended.

## Introduction

In 2019, there were ~2.9 million older people living with HIV (PWH) in low- and middle-income countries [1]. In the Eastern Europe and Central Asia region, the number of older PWH is rising. This is especially observed in Kazakhstan [2,3] among high-risk groups, including people who inject drugs and their sexual partners [4]. People who inject drugs account for 37% of HIV cases in Kazakhstan [5]. Given the success of antiretroviral therapies (ART), PWH are expected to live longer and achieve the life expectancy of people without HIV in Kazakhstan [6–9]. Globally and in Kazakhstan, there are increasing numbers of PWH age ≥ 40 years (y). Older age has an important influence on HIV treatment response and the occurrence of non-communicable diseases (NCDs) and associated morbidity [10]. The “aging’‘ of the HIV epidemic is mainly due to three factors: the success of ART; higher HIV incidence among older adults; and the often, unappreciated, observation that people aged ≥40 years engage in many risky behaviors (smoking, alcohol intake, and drug use) that lead to NCDs [11].

According to the Kazakh Scientific Center of Dermatology and Infectious Diseases, at the end of 2022, the estimated number of people in Kazakhstan who knew they had HIV was 31,233, and ~3,877 new cases were registered, with two times more males than females [12]. The majority, 82%, were among the working population aged 18–49 years. Sexual modes of transmission predominated and accounted for 75% of cases, and injection drug use mode of transmission accounted for 37% [13,14]. Data on older PWH are lacking [1,15–18].

To better understand aging-associated issues among older PWH in Kazakhstan, we conducted a first-ever cross-sectional study among PWH ≥ 40 years old who were enrolled at a major AIDS clinic in an urban center. We measured sociodemographic, health, behavioral, and HIV factors.

## Methods and materials

A cross-sectional study was conducted among a clinical sample of 150 PWH ≥ 40y at the Almaty City AIDS Center in Kazakhstan over a ~ 6-month period in 2023. Self-reported sociodemographic factors, health behaviors, personal and family history of risk factors and disease, mental and cognitive health, quality of life, and HIV factors were measured. Fasted blood samples were collected for plasma and serum measures. All surveys were conducted in the Russian language. Study data were managed using the Centers for Disease Control and Prevention KoBo Toolbox (https://www.kobotoolbox.org/).

*Sociodemographic factors* included date of birth, sex at birth (male, female), gender (man, woman, other), race (e.g., White, Black, other) and ethnicity (Kazakh, Russian, other), sexual orientation (sex partners), partnership status (married, single, divorced, separated, cohabitating, widowed), type and years of employment or unemployment, and maximum level of education attained. Education was divided into four levels:1) incomplete average education level (finished preschool, primary (1–4 grades) and lower secondary education (5–9 grades)); 2) full average education level (completed primary and lower secondary and upper secondary education (10–11 grades); and 3) professional college (colleges offer general and advanced academic as well as vocational education, lasting 3–4 years); and 4) undergraduate, incomplete and full higher education (undergraduate (Bachelor’s and Specialist degrees), postgraduate (Master’s and Doctoral degrees)). In addition, we queried about housing (own their home, live with a relative, live at a shelter, live outside, live at a boarding house).

*Personal and family medical history* included ever being diagnosed with or family history of cardiovascular disease, diabetes, high blood pressure, dementia, kidney disease, chronic lung disease, bone fracture, cancer, liver disease, thyroid disease, Acquired Immuno-deficiency Syndrome (AIDS), and other infectious diseases, such as hepatitis C virus (HCV), tuberculosis (TB), and other sexually transmitted infections (STI).

*Mental and cognitive health* assessments included depression (Patient Health Questionnaire, PHQ-9) [19,20] and anxiety (Generalized Anxiety Disorder, GAD-7) [21] symptoms; the Montreal Cognitive Assessment (MoCA) [22]; and World Health Organization Quality of Life (WHOQOL-26) questionnaire [23,24]. Clinically relevant symptoms were based on established cut-offs.

*Health behaviors* assessed included substance use, alcohol intake (type, intake frequency, and quantity), cigarette and other forms of smoking (number per day or per week), and sexual behaviors. Alcohol use disorder was assessed via the Alcohol Use Disorders Identification Test (AUDIT) [25], and substance use disorder via the Drug Use Disorders Identification Test (DUDIT) [26]. Regarding route of drug use, the only route of drug use queried in this sample was intravenous drug use (IDU).

*HIV factors* included self-reported ART use, with consideration for the Kazakh National clinical protocol for HIV treatment with two types of ART cocktails (preferable and alternative) approved by the Ministry of Health in Kazakhstan. The preferable ART regimen was TDF FTC DTG (tenofavir + emtricitabine + dolutegravir), and the alternative ART regimen was TDF FTC EFV (tenofavir + emtricitabine + efavirenz).

*Laboratory measures* included fasted EDTA plasma HIV viral load and CD4 count. These HIV biomarkers were measured in the nationally certified MPK laboratory, which provides clinical laboratory services for the Almaty City AIDS Center [27].

### Statistical analyses

Sociodemographic, medical history, mental health, health behavior, and HIV factors were characterized among all participants and by sex. Continuous variables were summarized using means and standard deviations (SD). T-tests were used to examine mean differences by sex. Categorical variables were summarized using frequencies and percents. Dichotomized outcome scores (e.g., yes/no moderate or high depression symptom burden) were examined in relation to categorical predictors using Chi-square analyses. Sex differences were explored. For those with ≤12 years of education, the MoCA score was increased by one point. Results were considered significant at p < 0.05. All statistical analyses were conducted using R Statistical Software (v4.3.1; R Core Team 2021).

### Ethical approval

The study protocol obtained institutional review board (IRB) approval by the Kazakh National Medical University Institutional Review Board. Written informed consent was obtained from each participant in accordance with the Declaration of Helsinki.

## Results

### Sociodemographic characteristics

This cross-sectional study recruited, enrolled and interviewed 150 PWH ≥ 40y (see Table 1). The participants’ average age was 51.6y (+/-10.4y; maximum 75.0y). Those aged > 70y (N = 5) were male. The median age was 49.0y (interquartile range, 11.5y). Stratified by sex, females were younger than males. The average age of females was 47.5y (+/-6.7y; range 40.0-65.0y; median 47.0y) and males were average age 56.8y (+/- 11.4y; range 41.0-75.0y; median 54.7y). Self-reported ethnicity included 22.7% Kazakh, 54.7% Russian and 19.3% Other or Unknown. Most of the males’ sex partners were females and most of the females’ sex partners were males. A lower educational level (incomplete average education level and lower secondary education) was observed among 76.0% (p = 0.001). Most participants (60.7%) lived in their own house.

**Table 1:** Sociodemographic characteristics of 150 PWH ≥ 40y by sex at the Almaty City AIDS Center.

Characteristics	Female N (%)	Male N (%)	Total N (%)	p-value for sex differences
	68 (45.3)	82 (55.7)	150 (100.0)	
Age (y)				0.020
40-49	45 (66.2)	37 (45.1)	82 (54.7)	
50-59	17 (25.0)	29 (35.4)	46 (30.7)	
60-69	6 (8.8)	11 (13.4)	17 (11.3)	
≥ 70	0 (0.0)	5 (6.1)	5 (3.3)	
Ethnicity				0.050
Kazakh	13 (19.1)	21 (25.6)	34 (22.7)	
Russian	41 (60.3)	46 (56.1)	87 (58.0)	
Other (Unknown)	14 (20.6)	15 (18.3)	29 (19.3)	
Marital status				0.010
married	18 (26.5)	38 (46.3)	56 (37.3)	
single	14 (20.6)	15 (18.3)	29 (19.3)	
divorced	12 (17.6)	17 (20.7)	29 (19.3)	
separated	0 (0.0)	1 (1.2)	1 (0.7)	
cohabitation	10 (14.7)	6 (7.3)	16 (10.7)	
widowed	11 (16.2)	4 (4.9)	15 (10.0)	
Sex partner				0.018
Male sex with male (MSM)	58 (85.3)	6 (7.3)	64 (42.7)	
Female sex with female	5 (7.4)	71 (86.6)	76 (50.6)	
sex with both	1 (1.4)	3 (3.7)	4 (2.7)	
Employment status				0.009
unemployed	23 (33.8)	20 (24.5)	43 (28.6)	
looking for a job	10 (14.7)	8 (9.9)	18 (12.0)	
unemployed	13 (19.1)	12 (17.6)	25 (16.6)	
retired	4 (5.9)	9 (10.9)	13 (8.7)	
disabled	4 (5.9)	2 (2.4)	6 (4.0)	
Employed	33 (48.5)	46 (56.1)	79 (52.7)	0.006
> 40h/week	11 (16.2)	15 (18.3)	26 (17.3)	
40 hours a week	18 (26.4)	21 (25.6)	39 (26.0)	
< 40 hours per week	4 (5.9)	10 (12.2)	14 (9.4)	
Educational status*				0.001
incomplete average	8 (11.7)	16 (19.5)	24 (16.0)	
full average	28 (41.2)	33 (40.2)	61 (40.7)	
professional college	15 (22.1)	13 (15.9)	28 (18.7)	
undergraduate	0 (0.0)	1 (1.2)	1 (0.7)	
incomplete higher education	2 (2.9)	1 (1.2)	3 (2.0)	
full higher education	15 (22.1)	18 (22.0)	33 (22.0)	
Life conditions/Housing				0.003
own their own home	41 (60.3)	50 (61.0)	91 (60.7)	
live with a relative	9 (13.2)	19 (23.2)	28 (18.6)	
live at a shelter	2 (2.9)	1 (1.2)	3 (2.0)	
live outside	0 (0.0)	1 (1.2)	1 (0.7)	
live at a boarding school	5 (7.4)	4 (4.9)	9 (6.0)	
HIV VL, mean(SD)	5903 (37645)	21648 (119025)	14510 (91654)	0.050
CD4 + count, mean(SD)	491 (269)	435 (190)	460 (230)	0.004

*Education System of Kazakhstan (incomplete average education level (finished preschool, primary (1–4 grades) and lower secondary education (5–9 grades)), full average education level (completed primary and lower secondary and upper secondary education (10–11 grades)), professional education at professional college (Colleges offer general and advanced academic as well as vocational education. The course of study is 3–4 years), higher education level (undergraduate (Bachelor’s and Specialist degrees), postgraduate (Master’s and Doctoral degrees)).

Sociodemographic characteristics by sex at birth among participants were similar, but there were differences in marital status (Table 1), with a higher proportion of males being married, and more widowed females than males. While over half of the participants were employed, employment status also differed by sex, with a higher proportion of females being unemployed at the time of the study visit compared to males.

### Behavioral factors

**Fig 1:**
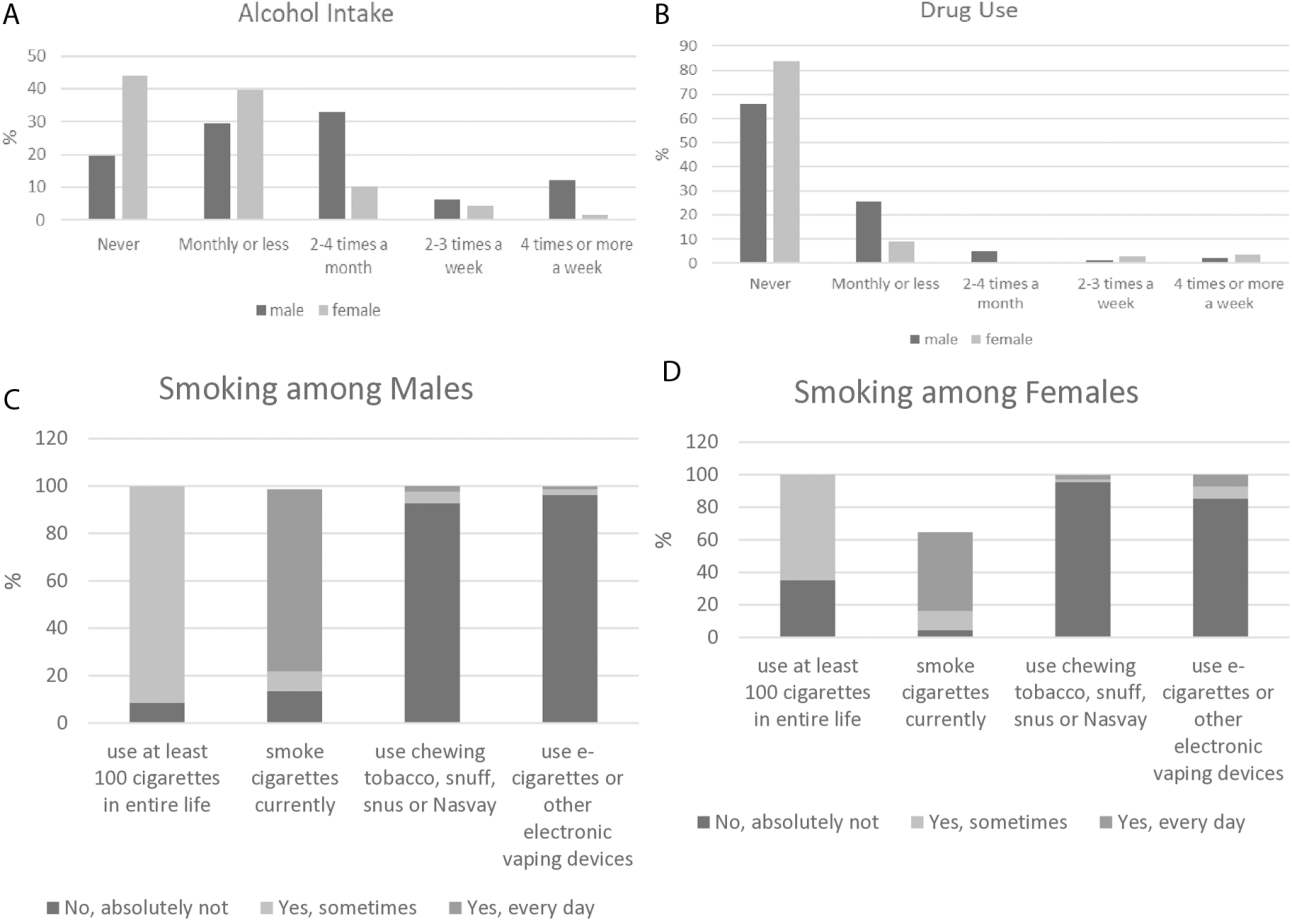
A. Alcohol intake among PLWH ≥ 40y by sex in Almaty, Kazakhstan. B. Drug use among PLWH ≥ 40y by sex in Almaty, Kazakhstan. C. Smoking among males with HIV ≥ 40y in Almaty, Kazakhstan. D. Smoking among females with HIV ≥ 40y in Almaty, Kazakhstan.

Alcohol intake and drug use, both frequency and duration among males and females are shown in Fig 1A and 1B. Monthly or less alcohol intake was higher among females (39.7%) compared to males (29.3%), but the highest frequency of alcohol intake (2–4 times per month) was observed among males (32.9% versus 10.3% among females, p = 0.001). The AUDIT score showed that more than 30% of participants never used alcohol, and 34% of participants were in the lowest risk category for alcohol consumption problems. Drug use was higher among males (25.6%) compared to females (8.8%, p = 0.050) via the DUDIT. The highest prevalence of alcohol consumption and drug use was observed among those aged 50-59y, 76.1% and 39.1%, respectively, compared to other age groups (40-49y, 60-69y).

The prevalence of smoking is shown in Fig 1C and 1D. Among males, 91.4% used at least 100 cigarettes in their lifetime, and 76.8% smoked cigarettes currently. Among females, 64.7% used at least 100 cigarettes in their lifetime, and 48.5% were current smokers (p = 0.001). Current smoking was more prevalent among those aged ≥60 years (86.3%) compared to other age groups.

### HIV factors

The average age at HIV diagnosis was 42.6y + /-10.4y (median 40y, IQR 14.5y; maximum age 66y). Of the different National clinical protocol ART regimens, TDF FTC EFV was taken by 38.3% of participants and 31.6% were taking the preferable regimen, TDF FTC DTG. The remaining participants were taking other ART regimens. The median number of years on ART was 6.0y.

Low CD4 cell count (<200 cells/mm^3^) was observed among 16.7% of participants, while 45% had medium (200–500 cells/mm^3^) and 38.3% high (above 500 cells/mm^3^) CD4 count. Furthermore, 76.7% participants had undetectable viral load (<50 copies/ml). Low CD4 cell count (<200 cells/mm^3^) was more prevalent among those aged 40-49y (18.1%) compared with older age groups.

### Personal and family history of disease

**Fig 2:**
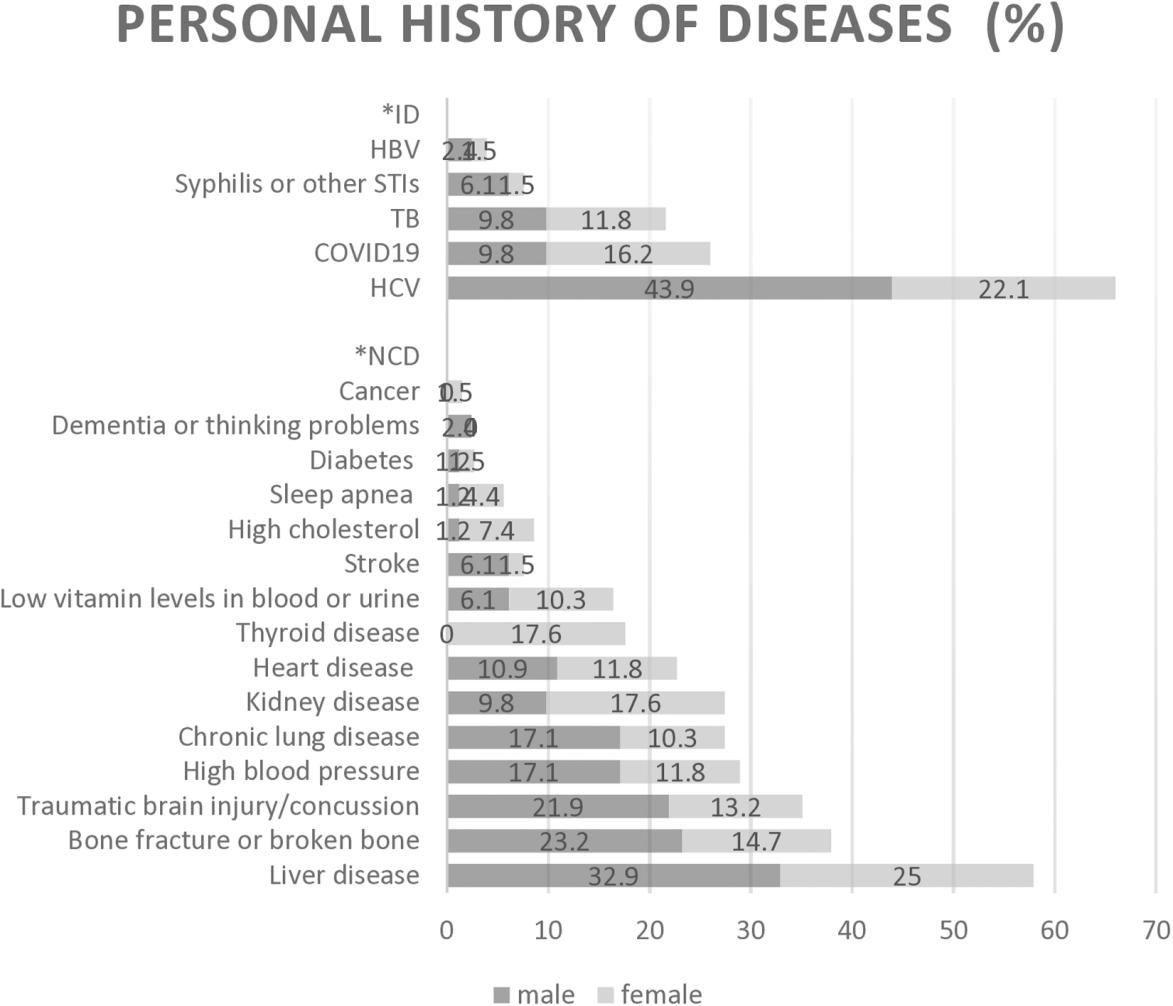
Self-reported personal history of diseases among males and females with HIV≥ 40y in Almaty, Kazakhstan.

Considering both infectious diseases (ID) and NCD, the most commonly reported personal histories of diseases are shown in Fig 2. Stratified by sex, there were differences in the distribution of some ID and NCD (p-values<0.05). The prevalence of chronic lung disease, high blood pressure, traumatic brain injury, bone fractures, liver diseases, HCV, and STI was higher among males compared to females (p-values=<0.05). However, the prevalence of heart disease, kidney disease, high level of cholesterol, and TB was higher among females compared to males (p-values=<0.05) (see Fig 2). Thyroid diseases were reported only by females and the prevalence was 17.6%.

Prevalence of heart disease, hypertension and chronic lung diseases differed by age group (p = 0.001). Among those aged 40-49y, self-reported history of stroke, kidney disease, high cholesterol, traumatic brain injury, bone fracture and thyroid disease was higher (p = 0.020) comparatively with the aged group 40-49y, and liver disease, TB and HCV were more prevalent among those aged 50-59y (p = 0.003) compared to other age groups.

Regarding family history, the most common self-reported conditions, reported by >10.0%, were high blood pressure (41.7%), followed by cardiovascular diseases (23.3%), and high blood glucose (12.6%). In addition, stroke (8.3%) and high cholesterol levels (3.3%) were reported. No participants reported a family history of dementia or Alzheimer’s disease.

### Mental health

The WHO QOL26 showed that 64.7% and 12.6% of participants had a good and very good quality of life, respectively, while 20.7% and 2.0% of participants had moderate and low quality of life, respectively. Physical and physiological health, environmental and social relationships among males and females were similar. (Table 2)

**Table 2:** Quality of life and mental health among PWH in Almaty, Kazakhstan.

	Very poor (%)	Poor (%)	Moderate (%)	High (%)	Very high (%)	P-value for sex differences 0.015
WHO QOL	0	2.0	20.7	64.7	12.6	
Physical health						
Male	0.0	1.2	13.4	39.0	46.4	
Female	0.0	4.4	27.9	38.3	29.4	
Psychological health						
Male	0.0	1.2	19.5	30.5	48.8	
Female	0.0	5.9	19.1	33.8	41.2	
Social relationships						
Male	1.2	7.3	19.6	57.3	14.6	
Female	2.9	7.4	26.5	51.5	11.7	
Environment						
Male	0.0	4.9	15.6	50.0	29.5	
Female	0.0	4.4	23.5	47.1	25.0	
**PHQ-9** Male	50.0	28.0	14.6	6.1	1.3	0.005
Female	33.8	35.2	16.2	7.4	7.4	
**GAD-7** Male	54.9	30.4	9.8	4.9	0.0	0.100
Female	47.1	33.8	8.8	10.3	0.0	

*Abbreviations: World Health Organization Quality of Life (WHO QOL), Patient Health Questionnaire-9 (PHQ-9), General Anxiety Disorder-7 (GAD-7).

High and very high depression symptom scores were more prevalent among females compared with males (p = 0.005). Higher levels of anxiety symptoms were also higher among females (10.3%) compared to males (4.9%). By age group, depression scores were higher among those aged 50-59y (8.7%), and anxiety scores were higher among those aged 40-49y (4.8%). Lower scores of depression and anxiety were observed among those aged ≥ 60y. Participants reporting high scores of depression or anxiety were more likely to live with relatives or renting apartments (p < 0.001), not finish secondary school (p < 0.001), be unemployed (p < 0.001), perceive their health to be poor (p = 0.010), and report poorer physical and mental health (p = 0.015). However, quality of life was overall good.

MoCA scores indicated that 61.9% were cognitively impaired (raw score <26; average 23.1). By sex, 62% of females had a low MoCA raw score <26; (average 24.1), while 72% of males had a raw score <26; average 23.5). Comparing those with MoCA < 26 versus ≥26, there were no differences in HIV viral load or CD4 + count. These results did not differ in all essential aspects whether one point was added to the score for PWH who reported ≤ 12y education.

## Discussion

Our pilot study was conducted in the southern Almaty region of Kazakhstan, in Almaty City, where 15.0% of PWH registered in the Republic of Kazakhstan live. This is the first descriptive cross-sectional study of older PWH described in Kazakhstan. Our study sample was enrolled from registered PWH patients at the Almaty City AIDS Center. As of 12/31/2019, there were 1382 PWH ≥ 50y registered at this AIDS center, with median age 55 years. Mortality occurred in almost half (48.6%) of PWH ≥ 50y between 2009–2019, and of those, 73.0% were males [28].

Alcohol intake and drug use are strongly associated with incidence and progression of HIV [29,30]. The prevalence of current smoking among PWH has been reported to be 2–3 times higher than that of the general population, which contributes to the higher incidence of non-HIV-related morbidity and mortality in PWH [31].

Prevalence of HCV, HBV, and TB co-infections among PWH has been reported to be higher due to behavioral risk factors [31,32]. In addition, having HIV can promote the progression of HCV- and HBV-associated liver disease, accelerate the development of fibrosis, and increase the risk of hepatic carcinoma [32]. In our study, 34.0% of PWH had HCV and this high prevalence of HIV/HCV co-infection highlights the necessity of regular screening for HCV infection among PWH, and increased availability of ART [33] and HCV cure measures. Kazakhstan fully adhered to the 2022 WHO recommendations to improve access to HCV cure and provide person centered care. The prevalence of HIV/TB coinfection is highest in Asian countries, including Kazakhstan [34], and the incidence of HIV/TB coinfection is increasing in Kazakhstan [35]. We observed HIV/TB coinfection in our sample.

Many studies have reported an association between older age and NCDs in PWH [36]. NCDs include cardiovascular disease, type 2 diabetes mellitus, hypertension, chronic kidney disease, and cancers. The prevalence of NCD risk factors including smoking, drug or alcohol use and lower socio-economic status, is high among older PWH [37,38]. Our findings demonstrated that self-reported history of liver disease, chronic lung disease, high blood pressure, traumatic brain injury, and bone fracture/broken bone was high. The combination of both HIV and NCD, as well as co-infections, lead to polypharmacy and difficulties in managing care for PWH. In addition, older PWH have most likely used more than one type of ART regimen over the course of their care, which complicates their medical history. In the 21^st^ century, the rapid increase of access to treatment for older PWH has led to healthcare challenges globally with an increasing aging population of PWH.

Cognitive impairment is common in people aging with HIV and can adversely affect health-related quality of life, access to care and treatment adherence. However, early cognitive impairment may be largely asymptomatic and cognitive performance is rarely assessed in the context of routine clinical care. In this study, we used the MoCA, which was available in the Russian language. In our sample, MoCA scores indicated that 61.9% were cognitively impaired, however, 98.0% of participants had a good quality of life and the prevalence of clinically relevant depression and anxiety symptoms was relatively low compared to other studies among PWH in this age group [39]. It may be that the MoCA is not an optimal global cognitive assessment tool in our population. This needs to be further studied.

Strengths of our study include a first ever sample of older PWH in Kazakhstan, relatively equal sex distribution, routine clinically-measured blood-based HIV biomarkers, data on comorbid infectious and chronic diseases, and lack of variable missingness. All data were collected in 2023, so have real-time relevance. However, there are also limitations. These data originate from a clinical sample of PWH who are patients at the Almaty City AIDS Center. Therefore, they are most likely to be mentally, cognitively and physically healthier compared to PWH who do not come to the clinic. The WHO QOL measure reflects this, and 84.1% of participants reported having at least a full average secondary education level, meaning they were for the most part a well-educated part of the Kazakh society. Age adjustments were not conducted due to the narrow age range. Finally, confirmation of self-reported personal and family medical history was not confirmed by medical record review, so there may be underreporting of medical history.The prevalence and specific types of aging-related comorbidities among PWH in Kazakhstan remain uncertain, despite that ~40% of PWH in the country are aged ≥ 40y [40]. Outcomes such as quality of life, depression, anxiety and cognition should be assessed in primary care HIV clinics. This will allow healthcare providers a more thorough understanding of the factors impacting older PWH and facilitate the provision of optimum person-centered care [41–43].

## Conclusion

Understanding and acknowledging sociodemographic and other health factors among older PWH in the Eastern Europe and Central Asia Region is crucial for healthcare providers and policy makers. This will provide evidence for the implementation of preventive measures, early screening, and interventions. Regular HCV and HBV screening, increased availability of ART and direct acting antivirals against HCV and HBV, as well as blood pressure and mental health monitoring should be implemented. In this study, the majority of PWH were of working age, employed, and reported good quality of life, however, sex differences were observed. Future prospective studies are recommended to further explore these disparities and guide comprehensive care strategies.
